# Evaluation of the Effectiveness of Nurse-Led Continence Care Treatments for Chinese Primary Care Patients with Lower Urinary Tract Symptoms

**DOI:** 10.1371/journal.pone.0129875

**Published:** 2015-06-15

**Authors:** Edmond P. H. Choi, Weng-Yee Chin, Cindy L. K. Lam, Eric Y. F. Wan, Anca K. C. Chan, Karina H. Y. Chan

**Affiliations:** Department of Family Medicine and Primary Care, The University of Hong Kong, Ap Lei Chau Clinic, Ap Lei Chau, Hong Kong; Supportive care, Early DIagnosis and Advanced disease (SEDA) research group, UNITED KINGDOM

## Abstract

**Background:**

The aim of this study was to evaluate whether community-based nurse-led continence care interventions are effective in improving outcomes for adult Chinese primary care patients with lower urinary tract symptoms (LUTS).

**Research Design and Subjects:**

A case-controlled intervention study was conducted. An intervention group of 360 primary care patients enrolled into a nurse-led continence care programme were recruited by consecutive sampling. A control group of 360 primary care patients with LUTS identified by screening were recruited from the waiting rooms of primary care clinics by consecutive sampling. Both groups were monitored at baseline and at 12 months.

**Measures:**

Outcome measures included symptom severity, health-related quality of life (HRQOL), self-efficacy, global health and self-reported health service utilization at 12-months. The effect of the continence care programme on symptom severity and HRQOL was assessed by the difference-in-difference estimation, using independent t-test and multiple liner regression. Chi-square test was used to compare the self-efficacy, global health and self-reported health service utilization between the two groups at 12-months.

**Results:**

After adjusting for baseline severity and socio-demographics, the intervention group had significant improvements in LUTS severity (P<0.05) and HRQOL (P<0.05). Improvements in the amount of urine leakage were not significantly different between the two groups. A higher proportion of subjects in the intervention group reported increased self-efficacy (43.48% vs. 66.83%), improved global health condition (17.74% vs. 41.5%), having doctor consultation (18.5% vs. 8.06), having medication due to LUTS (26.50% vs.11.29%) and having non-drug therapy due to LUTS (59.5% vs.9.68%).

**Conclusions:**

Community-based nurse-led continence care can effectively alleviate symptoms, improve health-related quality of life, and enhance self-efficacy and the global health condition of Chinese male and female primary care patients with LUTS.

## Introduction

As a result of population ageing and shifts in patient needs, there has been an increased demand for chronic disease management delivered in the community. Whilst the number of primary care doctors is limited, one solution has been to introduce allied health and nurse–led services to assist in health care delivery. A number of countries, namely the United Kingdom, Australia, Canada and the United States, have introduced multidisciplinary allied-health clinics, nurse practitioner, and nurse-led clinics into routine practice in primary care. Under certain circumstances, available evidence shows that this model for care delivery has the potential to reduce health costs without compromising quality of care [1,2]. Such delivery models are currently in their infancy in Asia, with little established evidence for its efficacy or effectiveness. In their 2008/2009 Policy Agenda, the Hong Kong SAR Government announced plans to enhance primary care and introduce new initiatives to strengthen support for chronic disease management. One of these was the introduction of Nurse and Allied Health Clinic Continence Care services (NAHC-CC), which have now been established within the government-funded General Outpatient Clinics (GOPC) of the Hospital Authority (HA) of Hong Kong.

Lower urinary tract symptoms (LUTS) are a highly prevalent that impacts quality of life. It is estimated that the worldwide prevalence of LUTS will rise from 45.2% in 2008 to 45.8% by 2018. LUTS affects both men and women. In order of frequency, the most common type of LUTS are storage symptoms, followed by voiding symptoms and post-micturition symptoms [3]. Urinary incontinence has been found to increase the risk of hospitalization and nursing home admission [4]. The degree of psycho-social morbidity associated with continence problems are highly variable and the patient’s perceived significance or impact on daily activities may have no direct relationship to the severity of the continence problem [5]. Systematic reviews have found that community-based services offering conservative continence care interventions can be effective in achieving improvements in urinary symptoms, continence-related knowledge, and quality of life [6–8]. Trained nurses can play an important role in providing assessments and conservative interventions such as pelvic floor muscle exercise, bladder training and fluid restriction for patients with LUTS [9].

A recent systematic review of community-based nurse-led continence services found that such care is effective [10], however many studies have had short follow-up periods (less than one year), have included only males, only females, or only patients with urinary incontinence, and none have been conducted in Asian settings. As LUTS are often caused by chronic conditions, the longer-term impact of interventions needs to be better evaluated to understand whether improvements in outcomes can be sustained. As voiding, storage and post-micturition symptoms almost always coexist in both females and males, it is important to assess whether such care can be effective for a broader range of patients [11]. Furthermore, as most studies to date have been conducted outside of Asia, there is little evidence available to demonstrate whether this model of care is transferable to non-Western healthcare settings.

### Aim

To help build the evidence base on nurse-led continence care, the aim of this study was to evaluate the effectiveness over 12 months of community-based nurse-led continence care services for male and female Chinese primary care patients with LUTS.

## Methods

### Subjects and sampling

This was a prospective, observational, case-control intervention study. Two cohorts of primary care patients with LUTS were recruited and monitored over 12 months (S1 Fig). One group received care from the NAHC-CC programme (‘intervention group’), whilst the other received usual care by their primary care physician (‘control group’).

Intervention group: All new patients referred to the NAHC-CC programme at one of four General Out-Patient Clinic (GOPC) locations distributed across Hong Kong during the study period were identified from the appointment list and screened for LUTS prior to their initial assessment by the nurse. All eligible subjects were invited to participate in the study.

Control group: Waiting room patients waiting to consult a doctor at one of four GOPC locations where NAHC-CC services were not available, were consecutively screened for LUTS prior to their medical consultation. All eligible subjects were invited to participate in the study.

For both groups, a modified International Consultation on Incontinence Questionnaire-Urinary Incontinence-Short Form (ICIQ-UI SF) questionnaire was used to screen for patient eligibility. Patients with scores ≥ 3 were considered to have LUTS [12]. Patients were excluded if they were aged < 18 years, could not understand Cantonese, or were too ill to give consent. Patients were also excluded if they had received continence care services from a nurse-led primary care clinic or a specialist clinic within the past one year for his/her LUTS.

Eligible patients were identified by trained field workers who approached the subjects in person to invite them to take part in a telephone survey. The aims, procedures and nature of the study were explained before consent was obtained. Subjects who consented were asked to provide their contact details and were subsequently contacted by trained interviewers who were blinded to the recruitment setting to administer the study instruments by telephone within two weeks of recruitment (baseline) and at 12-month after their baseline interview (follow-up). The questionnaires were read verbatim in a standardized way. Subjects were contacted using a computer-aided telephone interviewing system by the interviewers between 10:00 am and 10:30 pm on weekdays. A maximum of five attempts were made for unanswered lines.

#### NAHC-CC structure

NAHC-CC services are delivered by nurses with specialty related training related to urology or continence care, certified by the Hospital Authority. The nurses conduct the consultations, assessment and provide treatments in specially equipped consultation rooms located in the primary care Government-Outpatient clinics of the Hospital Authority, as part of a multi-disciplinary clinic with primary care doctors and nurse assistants. The main source of referrals is through the primary care physicians, clinic patients can also self-refer.

#### NAHC-CC Intervention

Patients enrolled in the NAHC-CC programme receive an initial assessment conducted by continence care nurses who conduct the history, physical examination and baseline investigations (uroflowmetry, pelvic floor muscle strength and post-void residual urine estimation). Subsequent interventions are protocol-based and include conservative measures tailored to each patient according to the type of LUTS being experienced. Interventions include pelvic floor muscle exercise, diet modification, bladder training and urethral massage for male patients:

- Pelvic floor muscle exercise involves repeatedly contracting and relaxing the pelvic floor muscles to improve the stiffness, the strength, the endurance, the coordination and the function of the muscles [13].
- Diet modification included evening fluid restriction and reduction in the consumption of fluids containing alcohol or caffeine [14].
- Bladder training is used to re-establish bladder control and restore a normal bladder patter by setting a schedule in which the patient may not use the toilet for voiding within a set period of time and to gradually increase the inter-voiding times. As the interval between visits to the toilet increases, the amount of urine that the bladder holds also increases [15].
- Urethral massage is used to eliminate post micturition dribble in male patients. The patient is instructed to place his fingers behind his scrotum after urination and to massages his bulbar urethra in a forwards and upwards direction so that the urine retained in the bulbar urethra can be released [15].

Patients subsequently receive one to two follow-up sessions provided at approximately 3–4 monthly intervals to monitor progress. If required, the continence care nurse may arrange referral to a specialist for patients who require advanced urological care or more specialized investigations such as urodynamic testing.

### Study Instruments

#### International Prostate Symptom Score (IPSS)

The IPSS comprises seven symptom questions and one health-related quality of life question. It asks about the severity of LUTS including incomplete bladder emptying, frequency of urination, intermittency, urgency, weak urine stream, straining and nocturia with higher scores indicating greater symptom severity. The psychometric performance of the Chinese IPSS has been evaluated in male and female patients with LUTS in Hong Kong primary care [16,17]. The IPSS total symptom score was the primary outcome measure for this study.

#### Modified International Consultation on Incontinence Questionnaire-Urinary Incontinence Short Form (Modified ICIQ-UI SF)

The original ICIQ-UI SF consists of four questions to assess the frequency (question 1) and amount of urinary leakage (question 2), the impact of urinary leakage on quality of life (question 3) and the perceived causes of urinary leakage (question 4), with higher scores indicating greater symptom severity and poorer health-related quality of life (HRQOL) [12]. For this study, the original ICIQ-UI was modified to broaden its applicability to all LUTS patients, and not just those with urinary incontinence (the modified questionnaire is shown in S1 File). A screening score ≥ 3 was used to define the presence of LUTS. Changes to the total sum score between baseline and 12 months were used a secondary outcome measure [18].

#### Modified Incontinence Impact Questionnaire–Short Form (IIQ-7)

The modified IIQ-7, which consists of 7 questions, was used to evaluate the impact of LUTS on HRQOL, with higher scores indicating poorer HRQOL. The psychometric performance of the modified Chinese IIQ-7 has been evaluated in male and female patients with LUTS in Hong Kong primary care [17,19].

#### Patient Enablement Instrument (PEI)

The PEI consists of six questions: able to cope with life, able to understand your illness, able to cope with your illness, able to keep yourself healthy, confident about your health and able to help yourself [20]. Subjects with PEI total score > 1 were considered to have enablement over the past 12-month period whilst those with PEI total score = 0 were considered to have no enablement over the period. The Chinese PEI has been validated in primary care patients in Hong Kong [20].

#### Global Rating of Change Scale (GRS)

The single item asks subjects to rate the change in their global health condition since the baseline interview [21]. The GRS was rated on a 5-point scale (+2 better, +1 a little bit better, 0 no change, -1 a little bit worse, -2 worse).

#### Self-reported health service utilization

Each subject was asked (i) whether he/she had consulted a doctor for urinary problems in the past 12 months, (ii) whether they had received medications for urinary problems in the past 12 months, (iii) whether he/she had surgery for urinary problems in the past 12 months, (iv) whether he/she had received non-drug therapy (dietary advice, pelvic floor muscle contract exercise and bladder training) for urinary problems in the past 12 months, (v) whether he/she had attended the emergency department for their urinary problems in the past six months and (vi) whether he/she had been admitted to hospital admission for urinary problems in the past 12 months.

The modified ICIQ-UI SF, IPSS and modified IIQ-7 were administered at baseline and 12-months. The PEI, GRS and self-reported health service utilization were only administered at 12-months. The Chinese versions of the instruments were used.

### Sample size estimation

The sample size for the evaluation of the difference in the mean change of the IPSS total symptom score after 12- month between groups with moderate effect size 0.3 was estimated. A sample size of 176 subjects in each group was needed in order to have 80% power and 5% level of significance by independent t-test. With 30% attrition rate at each time point, the minimum number of subjects in each group at baseline is 360.

### Statistical analyses

Descriptive statistics were used to calculate the baseline characteristics of socio-demographics and clinical outcomes at baseline and 12-month. For calculation of the mean change in outcomes (ICIQ-UI SF, IPSS and IIQ-7 scores), within-subject changes 12-months after baseline were analyzed by paired t-test. Unadjusted difference-in-difference estimation in outcomes between groups were reported and analyzed by independent t-test. The amount of urine leakage (the ICIQ-UI SF question 2) was further analyzed in patients with urinary incontinence by gender groups. Adjusted difference-in-difference was used to further analyze the outcomes including ICIQ-UI SF, the IPSS and the IIQ-7. Adjusted difference-in-difference estimation between groups was assessed by multiple liner regressions by the adjustment of confounding factors (socio-demographics, baseline symptom severity and the respective outcomes at baseline). Chi-square test was used to assess the difference in PEI, GRS and self-reported health service utilization at 12-months between intervention and control groups.

All statistical analyses were performed using STATA Version 13.0 (StataCorp LP. College Station, Texas, U.S.). All significance tests were two-tailed and findings with a p-value less than 0.05 were considered statistically significant.

### Ethics approval

The study protocol of the present study was approved by the institutional review boards: the University of Hong Kong/Hospital Authority Hong Kong West Cluster (UW12-558), Hong Kong East Cluster (HKEC-2010-095), Kowloon West Cluster (KW/EX/10-149(34–16)) and Kowloon East Cluster (KC/KE-10-0209/ER-3). Participants provided their written informed consent to participate in this study. The ethics committees approved this consent procedure and this study before enrollment of participants started.

## Results

A flowchart outlining subject recruitment, follow-up and analysis is shown in S1 Fig. Overall, 720 subjects (360 subjects in each group) with LUTS were recruited from 4 March 2013 to 16 July 2013. Of these, 406 subjects (200 intervention subjects and 206 controls) completed the 12-month telephone interview. Twenty subjects in the control group were excluded from analysis as they had joined the NAHC-CC programme for their LUTS subsequent to the baseline interview.

Table 1 shows the baseline socio-demographic characteristics of the study subjects. The mean age of subjects in the control group was slightly older than the intervention subjects. There were more females and subjects who were not in active employment in the control group. Subjects in the intervention group had significantly higher ICIQ-UI SF score, IPSS quality of life score and IIQ-7 score than subjects in control group. There was no significant difference in IPSS total symptom score between groups.

**Table 1:** Demographic and clinical characteristics of subjects included in the NAHC-CC effectiveness study.

	Intervention group	Control group	P-value
	(N = 200)	(N = 186)	
**Demographic characteristics**			
Age (mean ± SD)	60.30±11.38 (200)	63.22±9.34 (186)	0.006#
Gender (%, n)			0.037*
Female	53.50% (107)	63.98% (119)	
Male	46.50% (93)	36.02% (67)	
Marital status (%, n)			0.366
Married	79.00% (158)	73.66% (137)	
Single	4.00% (8)	3.23% (6)	
Widowed	12.00% (24)	13.98% (26)	
Divorced	5.00% (10)	9.14% (17)	
Employment status (%, n)			0.001#
Working	43.00% (86)	27.42% (51)	
Not working	57.00% (114)	72.58% (135)	
Household income (%, n)			0.11
< $20,000	29.00% (58)	19.89% (37)	
≥ $20,000	60.50% (121)	69.35% (129)	
Missing	10.50% (21)	10.75% (20)	
Smoking status (%, n)			0.158
Never	74.00% (148)	77.42% (144)	
Ex-smoker	21.00% (42)	15.05% (28)	
Current smoker	1.00% (2)	3.76% (7)	
Refused to answer	4.00% (8)	3.76% (7)	
Drinking status (%, n)			0.272
Never	48.00% (96)	53.23% (99)	
Ex-drinker	10.50% (21)	7.53% (14)	
Current drinker	38.00% (76)	32.80% (61)	
Refused to answer	3.50% (7)	6.45% (12)	
**Clinical characteristics** (mean ± SD)			
ICIQ-UI SF total score	8.17±3.83 (199)	7.14±3.81 (186)	0.009#
IPSS total symptom score	11.49±5.90 (200)	10.49±6.70 (186)	0.122
IPSS quality of life score	3.72±1.71 (200)	2.94±1.81 (186)	<0.001#
IIQ-7 score	3.77±4.37 (200)	3.00±3.91 (186)	0.071

SD: standard deviation

ICIQ-UI SF: International Consultation on Incontinence Questionnaire-Urinary Incontinence-Short Form

IPSS: International Prostate Symptom Score

IIQ-7: Incontinence Impact Questionnaire

* Significant with p-value < 0.05

# Significant with p-value < 0.01

The results of unadjusted difference-in-difference estimation of the IPSS total symptom, IPSS HRQOL, ICIQ-UI SF and IIQ-7 scores between groups were shown in Table 2. With regard to within-group difference, the IPSS total symptom score, IPSS quality of life score, ICIQ-UI SF total score and the IIQ-7 had significant improvement with effect sizes >0.3 in intervention group. In the control group, the IPSS total symptom score, IPSS quality of life score and the IIQ-7 also had significant improvement but the effect sizes were only trivial. We further examined the change in score of the ICIQ-UI SF question 2 only in subjects with urinary incontinence by gender group. Subgroup analysis found that the amount of urinary leakage significantly improved in female subjects in both groups but not in male subjects. There was a significant difference in the mean change in score of the IPSS total symptom, IPSS HRQOL and IIQ-7 but not the ICIQ-UI SF total scores between two groups. Furthermore, subgroup analysis found that there was no significant difference in the mean change in score of the ICIQ-UI SF question 2 between the two females groups.

**Table 2:** Comparison of clinical characteristics of subjects between two groups at baseline and 12-month follow-up.

	Intervention group				Control group				Difference in change (intervention–control groups)	P-value
	(N = 200)				(N = 186)					
	Baseline	12 months	Paired Difference	Effect Size	Baseline	12 months	Paired Difference	Effect Size		
IPSS total symptom score (min 0—max 35)	11.55±5.91	8.66±6.09	-2.89**	0.48	10.49±6.70	9.34±7.44	-1.15**	0.16	-1.74	0.002**
IPSS quality of life score (min 0—max 6)	3.72±1.71	2.71±1.82	-1.01**	0.57	2.94±1.81	2.61±1.75	-0.33**	0.19	-0.68	<0.001**
ICIQ-UI SF total score (min 0—max 21)	8.16±3.83	5.35±4.40	-2.81**	0.68	7.14±3.81	4.60±3.81	-2.54**	0.67	-0.28	0.486
IIQ-7 score (min 0—max 21)	3.77±4.37	2.45±3.34	-1.32**	0.34	3.00±3.91	2.71±3.69	-0.29	0.08	-1.03	0.002**
	All subjects with urinary incontinence				All subjects with urinary incontinence					
	(N = 120)				(N = 134)					
ICIQ-UI SF Q2 score (min 0—max 6)	1.90±1.24	1.33±1.28	-0.57**	0.45	1.89±0.93	1.37±1.08	-0.52**	0.52	-0.04	0.788
	Male subjects with urinary				Male subjects with urinary incontinence					
	(N = 31)				(N = 26)					
ICIQ-UI SF Q2 score (min 0—max 6)	1.55±1.23	1.35±1.31	-0.19	0.16	1.92±1.20	1.46±1.21	-0.46	0.38	0.27	0.422
	Female subjects with urinary incontinence				Female subjects with urinary incontinence					
	(N = 89)				(N = 108)					
ICIQ-UI SF Q2 score (min 0—max 6)	2.02±1.22	1.33±1.28	-0.70**	0.55	1.89±0.86	1.35±1.05	-0.54**	0.56	-0.16	0.401

** Significant at a level of 0.01 by paired t-test or independent t-test as appropriate

Results of the adjusted difference-in-difference estimation of scores between groups are shown in Table 3. After adjusting for socio-demographics and corresponding score at baseline, the intervention group showed a greater reduction in LUTS severity as measured by the IPSS, and better improvements in HRQOL as measured the IIQ-7. Moreover, more severe LUTS at baseline were associated with greater reduction in LUTS severity at 12-month follow-up. Poorer HRQOL and less severe LUTS at baseline were associated with greater improvement in HRQOL at 12 months as measured by both the IPSS and the IIQ-7.

**Table 3:** Adjusted difference-in-difference by multiple linear regressions.

	Change in ICIQ-UI SF total score			Change in IPSS total symptom score			Change in IPSS quality of life score			Change in IIQ-7 score		
	(N = 316)			(N = 317)			(N = 317)			(N = 318)		
	R2 = 29.4%, Adj. R2 = 26.8%			R2 = 17.0%, Adj. R2 = 14.3%			R2 = 28.6%, Adj. R2 = 26.0%			R2 = 39.7%, Adj. R2 = 37.5%		
Independent variables ^†^	Coef.	95% CI	P-value	Coef.	95% CI	P-value	Coef.	95% CI	P-value	Coef.	95% CI	P-value
***Treatment***												
Intervention group	0.057	(-0.708,0.823)	0.883	-1.403*	(-2.555,-0.251)	0.017*	-0.337	(-0.681,0.008)	0.055	-0.726*	(-1.347,-0.105)	0.022*
(vs. control group)												
***Patient characteristics***												
Age	-0.046*	(-0.092,-0.001)	0.044*	-0.024	(-0.092,0.044)	0.481	-0.023*	(-0.043,-0.003)	0.023*	-0.041*	(-0.078,-0.004)	0.028*
Male	-0.47	(-1.528,0.588)	0.383	2.096*	(0.498,3.694)	0.010*	0.039	(-0.429,0.507)	0.87	-0.14	(-0.998,0.719)	0.749
(vs. female)												
Married	0.529	(-0.399,1.456)	0.263	0.299	(-1.104,1.702)	0.675	-0.226	(-0.639,0.186)	0.282	0.389	(-0.361,1.139)	0.308
(vs. unmarried)												
Employed	-0.436	(-1.348,0.477)	0.348	-0.434	(-1.808,0.939)	0.534	-0.052	(-0.455,0.352)	0.802	-0.446	(-1.183,0.291)	0.235
(vs. unemployed)												
Household income ≥ $20,000	0.372	(-0.499,1.244)	0.401	-0.158	(-1.472,1.157)	0.813	-0.029	(-0.413,0.355)	0.883	-0.219	(-0.934,0.497)	0.548
(vs. < $20,000)												
Current smoker	0.85	(-1.503,3.203)	0.478	-0.038	(-3.599,3.522)	0.983	-0.062	(-1.103,0.979)	0.907	1.627	(-0.283,3.538)	0.095
(vs. nonsmoker)												
Current drinker	0.733	(-0.058,1.524)	0.069	0.162	(-1.032,1.356)	0.789	0.105	(-0.244,0.454)	0.554	-0.491	(-1.131,0.150)	0.133
(vs. nondrinker)												
Urinary incontinence	0.557	(-0.399,1.512)	0.253	-0.614	(-2.040,0.811)	0.397	0.000	(-0.417,0.417)	0.999	-0.06	(-0.834,0.713)	0.878
(vs. no urinary incontinence)												
***Baseline scores***												
ICIQ-UI SF total score	-0.553*	(-0.660,-0.446)	< 0.001#	Not considered			Not considered			Not considered		
IPSS total symptom	0.176*	(0.111,0.241)	< 0.001#	-0.308*	(-0.398,-0.219)	< 0.001#	0.058*	(0.028,0.087)	< 0.001#	0.102*	(0.044,0.159)	0.001#
IPSS quality of life score	Not considered			Not considered			-0.528*	(-0.632,-0.423)	< 0.001#	Not considered		
IIQ-7 score	Not considered			Not considered			Not considered			-0.539*	(-0.627,-0.452)	< 0.001#
Constant	1.602	(-1.646,4.850)	0.332	3.002	(-1.851,7.855)	0.224	2.159*	(0.731,3.586)	0.003*	2.988*	(0.375,5.601)	0.025*

* Significant with p-value < 0.05

# Significant with p-value < 0.01

^†^ Variable in brackets is the reference category for independent variables.

The result of the patient enablement index (PEI) and global rating of change scale (GRS) at 12-months is shown in Table 4. Compared with the control group, more subjects in the intervention group had improved self-efficacy (43.48% vs. 66.83%) as measured by the PEI and improved general health condition (17.74% vs. 41.5%) as measured by the GRS.

**Table 4:** PEI and GRS of subjects included in the NAHC-CC effectiveness study after 12 months.

	Intervention group	Control group	P-value
	(N = 200)	(N = 186)	
**PEI score (%, n)**			<0.001*
Enabled	66.83% (133)	43.48% (80)	
Not enabled	33.17% (66)	56.52% (104)	
**GRS (%, n)**			<0.001*
Improved	41.50% (83)	17.74% (33)	
Stable	39.50% (79)	51.61% (96)	
Worse	19.00% (38)	30.65% (57)	

PEI: Patient Enablement Instrument

GRS: Global Rating Scale

* Significant with p-value < 0.001

Table 5 shows the self-reported health service utilization in the intervention and control groups. More subjects in the intervention group reported having received doctor consultations due to LUTS in the past 12 months (18.5% vs. 8.06%), having medication due to LUTS in the past 12 months (26.5% vs. 11.29%), having non-drug therapy (such as pelvic floor muscle exercises and bladder training) for urinary incontinence (59.5% vs. 9.68%)

**Table 5:** Self-reported service utilization of subjects included in the NAHC-CC effectiveness study.

	Intervention group	Control group	P-value
	(N = 200)	(N = 186)	
Doctor consultation due to urinary problems in the past 12 month			0.003#
No	81.50% (163)	91.94% (171)	
Yes	18.50% (37)	8.06% (15)	
Medication due to urinary problems in the past 12 months			<0.001#
No	73.50% (147)	88.71% (165)	
Yes	26.50% (53)	11.29% (21)	
Operation due to urinary problems in the past 12 months			0.334
No	99.50% (199)	100.00% (186)	
Yes	0.50% (1)	0.00% (0)	
Non-drug therapy due to urinary problems in the past 12 months			<0.001#
No	40.50% (81)	90.32% (168)	
Yes	59.50% (119)	9.68% (18)	
Dietary advice	22.69% (27)	11.11% (2)	0.262
Pelvic floor muscle contraction exercise	88.24% (105)	66.67% (12)	0.016*
Bladder training	29.41% (35)	5.56% (1)	0.032*
AED attendance due to urinary problems in the past six months			0.334
No	99.50% (199)	100.00% (186)	
Yes	0.50% (1)	0.00% (0)	
Hospital admission due to urinary problems in the past 12 months			0.334
No	99.50% (199)	100.00% (186)	
Yes	0.50% (1)	0.00% (0)	

* Significant with p-value < 0.05

# Significant with p-value < 0.01

## Discussion

This study examined the impact of community-based nurse-led continence care interventions on Chinese primary care patients with LUTS. This is one of the first studies to evaluate whether the model of community-based nurse-led continence care is effective in an Asian public sector healthcare setting. The 12-month cohort design allowed evaluation of effectiveness as well as sustainability of interventions in improving clinical outcomes.

Our key finding was that whilst both groups experienced statistically significant improvements in the amount of urine leakage, IPSS total symptom scores and IPSS HRQOL scores between baseline and 12 months, improvements observed in the intervention group were significantly greater than those in the control group. Improvements observed between baseline and 12 months indicates that even without specific LUTS interventions, many patients can naturalistically improve. This may be a result of treatments provided during usual medical care [22], or patients may simply learn over time how to adjust their behaviors and adapt to the LUTS. It should be noted that effect sizes were very small in the control group (total symptom score: 0.16; HRQOL score: 0.19), and although they were statistically significant, may not be large enough to be considered clinically significant.

The magnitude of the IPSS total symptom score reduction in the intervention group (2.89 out of 35 points) in this study setting appears to be smaller than score reductions reported in a previous study males with LUTS (8.55 out of 35points) [23], however, subjects in that study had a more severe spectrum of LUTS and cannot be directly compared to our study population of primary care patients with a milder spectrum of disease [23]. The relatively modest effect of continence care interventions in our study could be due to the lower baseline severity and subsequently a smaller scope for improvement. Even after controlling for baseline characteristics, we found that the intervention group had significantly greater improvement in LUTS severity and HRQOL than the control group at the 12-month follow-up. This supports our hypothesis that nurse-led conservative interventions are effective in improving outcomes for patients with LUTS when compared to usual care.

Another important finding in this study was that the intervention group, but not the control group reported significant improvements in HRQOL as measured by the IIQ-7 over 12 months. This may be an outcome of treatment interventions which included intensive lifestyle modification with the aim of enhancing patient self-empowerment, which in turn helped to substantially alleviate the negative impacts of LUTS on their daily lives.

A subgroup analysis of the effect of the programme on urine leakage as measured by the ICIQ- UI SF found that in both intervention and control groups, female subjects reported significant improvements in the amount of urine leakage. We hypothesized that female subjects might be more willing to report the presence of urinary symptoms to their healthcare provider or social circle, and as a result may have received advice or treatments for incontinence during usual care or in the course of their usual social activities. Further study is needed to explore the coping mechanism of female patients with urinary incontinence problems. On the contrary, lower urinary tract problems are often linked to masculinity and reduced sexual competency in males [24]. As a consequence, males with urinary incontinence might be more reluctant to discuss the problems with others. Further qualitative studies to explore the meaning of LUTS in Chinese patients are needed. There are other possible reasons for the insignificant improvement of urine leakage in male patients. First, the amount of urine leakage at baseline was relatively mild (1.55 out of 6 points in intervention group) in males, which allows little room for improvement. Second, the sample size of the male patients with urinary incontinence in both cohorts was small, which might affect the power and significance of the findings and further studies with a larger sample of males with urinary incontinence is needed to confirm this finding. Third, as many males with LUTS will have prostatic enlargement, a continuing decline is more likely to be expected if no interventions are provided [25].

At the 12-month follow up, the NAHC-CC programme, intervention subjects reported increased self-efficacy and improved general health condition. Similar to studies in other chronic disease conditions, we found that self-management strategies provided by continence care nurses can increase self-efficacy in illness management for patients with LUTS [26]. The immediate effect of self-management is to help patients regain the sense of control [27] so that they can have more control over their lives and illness [28]. Moreover, increase in self-efficacy can result in better adherence with intervention and improved health status [29–31]. It was reassuring to see that these self-reported outcomes were still reported 12-months after the baseline assessment.

This study had a number of notable limitations. First, a randomized control trial was not conducted and unknown confounders may have biased the results. However, this was a naturalistic study which is more easily translatable to real clinical care [32]. Second, all clinical outcomes were patient-reported outcomes, and may be affected by recall bias. Objective clinical parameters such as uroflowmetry should be included as outcome measures in future studies. Third, participation in the NAHC–CC programme was voluntary and it is likely that patients with better motivation to control their LUTS would agree to enter the programme. Although patients are charged a fee of HKD45 (USD 5.80) per consultation, this is only slightly greater than the fee for a routine GOPC consultation is HKD25 (USD 3.20) and is not a significant barrier to joining the programme. Fourth, our study was conducted in a public-sector primary care setting and the findings may not be applicable to all Chinese patients with LUTS, such as those found in specialist settings. Fifth, data was not collected on the number of continence care sessions and general medical consultations over the 12 months which would be important for assessing the cost effectiveness of the programme. This information is also needed to explore the association between amount of improvement and amount of services provided.

## Conclusions

This longitudinal observation study found that a structured nurse-led continence care programme is effective in alleviating symptoms and improving HRQOL at 12-months in Chinese patients with LUTS, but is less effective in improving urinary incontinence. Different from other studies on self-management programmes for LUTS, this evaluation study investigated the effect of nurse-led interventions for LUTS patients in a pragmatic primary care setting, which provides better translational evidence. A further study focusing on the long term effects of the programme is currently being conducted.

## Supporting Information

S1 Fig Subject recruitment and follow up flowchart. Overall, 720 subjects (360 subjects in each group) with LUTS were recruited. Of these, 406 subjects (200 intervention subjects and 206 controls) completed the 12-month telephone interview. Twenty subjects in the control group were excluded from analysis as they had joined the NAHC-CC programme for their LUTS subsequent to the baseline interview
(PDF)

S1 File Screening Questionnaire: Modified ICIQ-UI SF. (PDF)
