# Supplementary material for: Evaluation of the Effectiveness of Nurse-Led Continence Care Treatments for Chinese Primary Care Patients with Lower Urinary Tract Symptoms
Source: PLoS One. 2015 Jun 15;10(6):e0129875. doi: 10.1371/journal.pone.0129875 (PMC4467983; doi:10.1371/journal.pone.0129875)
Supplement: S1 Fig — Overall, 720 subjects (360 subjects in each group) with LUTS were recruited. Of these, 406 subjects (200 intervention subjects and 206 controls) completed the 12-month telephone interview. Twenty subjects in the control group were excluded from analysis as they had joined the NAHC-CC programme for their LUTS subsequent to the baseline interview (PDF) [file pone.0129875.s001.pdf]

S1 Fig: Subject recruitment and follow up flowchart

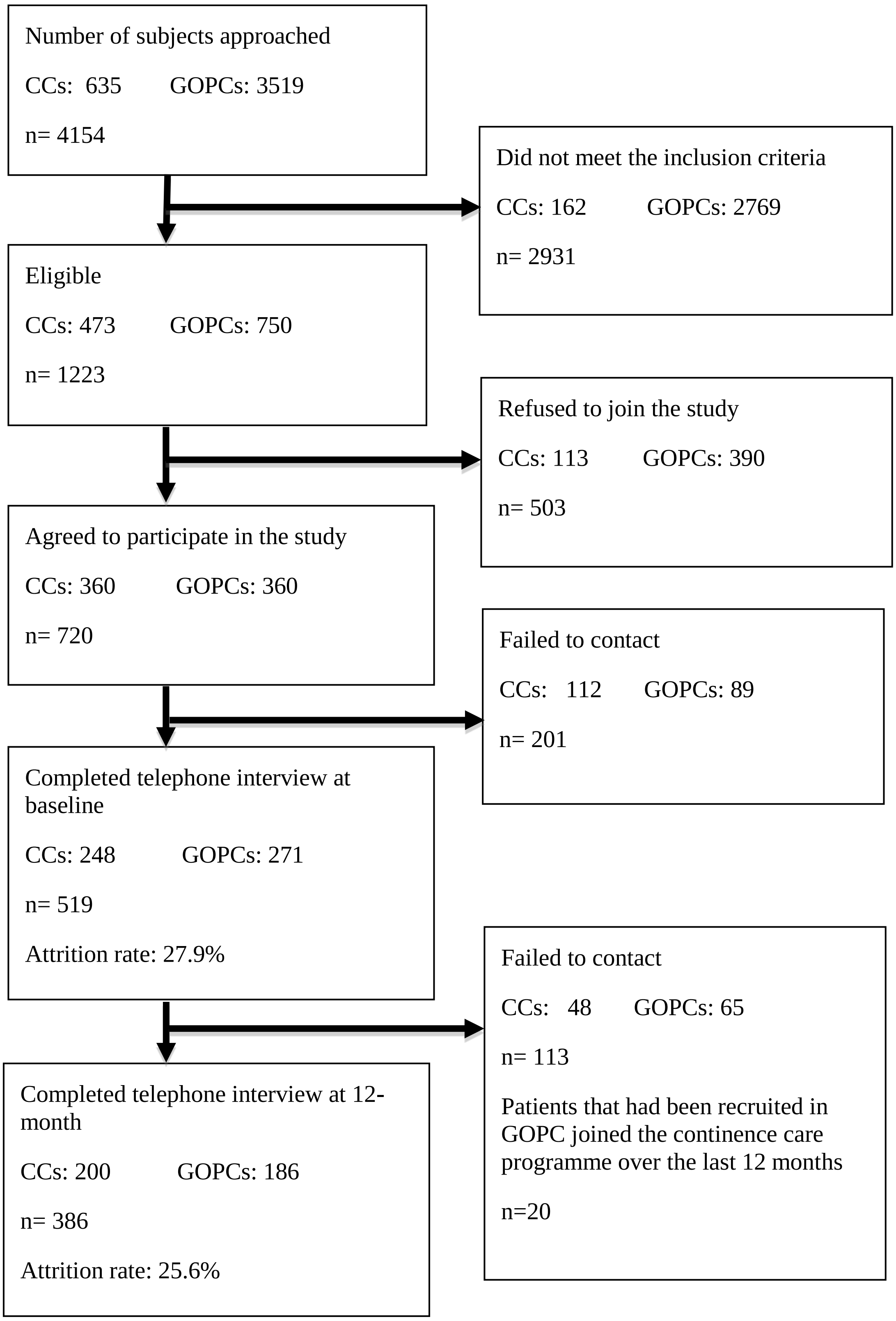

CCs: Continence care clinics

GOPCs: General outpatient clinics
