# Supplementary material for: Evaluation of the Effectiveness of Nurse-Led Continence Care Treatments for Chinese Primary Care Patients with Lower Urinary Tract Symptoms
Source: PLoS One. 2015 Jun 15;10(6):e0129875. doi: 10.1371/journal.pone.0129875 (PMC4467983; doi:10.1371/journal.pone.0129875)
Supplement: S1 File — (PDF) [file pone.0129875.s002.pdf]

S1 File: Screening questionnaire (Modified ICIQ-UI SF)

1. How often do you have urinary problems *?

Never	☐	0
About once a week or less often	☐	1
Two or three times a week	☐	2
About once a day	☐	3
Several times a day	☐	4
All the time	☐	5

2. We would like to know how much urine you think leaks.
How much urine do you usually leak (whether you wear protection or not)?

None	☐	0
A small amount	☐	2
A moderate amount	☐	4
A large amount	☐	6

3. Overall, how much do urinary problems * interfere with your everyday life?
Please ring a number between 0 (not at all) and 10 (a great deal)

0 1 2 3 4 5 6 7 8 9 10

Total Score: 1+2+3:____

Urinary problems: intermittent stream, incomplete bladder emptying, weak stream, straining, nocturia, urgency, frequency and incontinence etc.
